# Novel Indices to Improve the Diagnostic Ability of Nocturnal Oximetry in Children with OSAS

**DOI:** 10.3390/children10030453

**Published:** 2023-02-25

**Authors:** Panagiota Pappa, Konstantinos Kourelis, Anastasios Goulioumis, Magdalene Tsiakou, Panagiotis Plotas, Aris Bertzouanis, Ilias Theodorakopoulos, Konstantinos Mourtzouchos, Michael B. Anthracopoulos, Athanasios Asimakopoulos, Sotirios Fouzas

**Affiliations:** 1 School of Medicine, University of Patras, 26500 Patras, Greece; 2 Department of Otorhinolaryngology, ‘Karamandaneio’ Children’s Hospital, 26331 Patras, Greece; 3 Laboratory of Primary Health Care, School of Health Rehabilitation Sciences, University of Patras, 26500 Patras, Greece; 4 Pediatric Pulmonology Research Group, Pediatric Respiratory Unit, University Hospital of Patras, 26504 Patras, Greece; 5 Department of Electrical and Computer Engineering, Democritus University of Thrace, 67132 Xanthi, Greece

**Keywords:** obstructive sleep apnea syndrome, sleep-disordered breathing, nocturnal oximetry

## Abstract

Nocturnal pulse oximetry (NOx) is an alternative diagnostic test for obstructive sleep apnea syndrome (OSAS) in childhood yet with variable diagnostic performance. Our aim was to apply advanced signal analysis to develop novel and more accurate NOx indices. We studied 45 children aged 3–10 years who underwent adenotonsillectomy for adenotonsillar hypertrophy and OSAS symptoms. Participants performed NOx before and three months after surgery, and the changes in McGill oximetry score (MOS), oxygen desaturation ≥3% index (ODI3), and the novel parameters—cumulative saturation area (CSA) and oxygen saturation sample entropy (SSE)—were assessed. There was a significant improvement (*p* < 0.001) in all NOx indices. When pre- and post-adenotonsillectomy NOx recordings were compared, the MOS had an area under the curve (AUC) of 0.811 with 63.2% sensitivity and 100% specificity at a cutoff >1. The AUC of ODI3 was 0.994, with 97.8% sensitivity and 91.1% specificity at a cutoff of >3.6 events per hour. The CSA and SSE had an AUC of 1.00, with 100% sensitivity and specificity at a cutoff of >293 and >0.99, respectively. We conclude that the herein-introduced indices—CSA and SSE—hold promise in improving the diagnostic ability of NOx in children suspected of OSAS.

## 1. Introduction

Obstructive sleep-disordered breathing (SDB) in childhood refers to a spectrum of disorders characterized by upper airway dysfunction during sleep due to increased local airway resistance and pharyngeal collapsibility [1,2]. The syndrome is expressed clinically by habitual snoring (>3 nights per week) and various degrees of respiratory effort that may disrupt the physiological sleep pattern [1,3,4]; non-specific sleep-related symptoms such as teeth grinding (bruxism) may also be present [5]. Obstructive sleep apnea syndrome (OSAS), the most severe form of SDB, is characterized by recurrent episodes of partial or complete upper airway obstruction that result in snoring, hypopneas, obstructive or mixed apneas, intermittent hypoxemias, and sleep fragmentation [1,3,4]; it is estimated to occur in 2–3% of school-aged children [6].

Polysomnography (PSG) is the gold standard for OSAS diagnosis and severity stratification [1,3]. PSG involves the overnight, attended, in-laboratory recording of various physiological parameters, including electroencephalography, electrooculography, nasal and oral airflow, abdominal and chest wall movements, oxygen saturation by pulse oximetry (SpO_2_), partial pressure of carbon dioxide, electrocardiography, submental and leg electromyography, and body movements, as well as video recording [7]. The number of mixed (obstructive and central) apneas and hypopneas per hour, called the apnea–hypopnea index (AHI), is the most used parameter [1,3,7]. Generally, an AHI ≥ 1 episode per hour in a child with relevant symptoms is considered indicative of OSAS [1,3]; an AHI of 1–5 episodes per hour indicates mild OSAS; while an AHI >5 corresponds to moderate-to-severe disease that is more likely to benefit from adenotonsillectomy [1,8,9]. Nevertheless, PSG remains a laborious, time-consuming, and expensive testing modality that is often considered inconvenient by children and their parents [1,10,11]. Moreover, the availability of pediatric sleep laboratories even in countries with advanced health systems is limited [12,13], which has led to the quest for less complex and more convenient testing modalities [1,14,15].

Nocturnal pulse oximetry (NOx) has been proposed as a simpler test for OSAS diagnosis when PSG is not available [15,16]. This method has several advantages: it is easy to apply and interpret, has low cost, does not require close attendance, and can be performed at the patient’s home under usual sleep conditions [15,16]. NOx relies on the identification of abrupt SpO_2_ drops (desaturations) that accompany obstructive apneas and hypopneas during REM and N2 sleep [4]. Specific NOx parameters, such as the oxygen desaturation indices and the McGill criteria, which assess the frequency, magnitude, and pattern of desaturation events, have been used to confirm the presence of OSAS in symptomatic children and estimate its severity [1,15,16,17]. However, mild episodes of upper airway obstruction may not necessarily result in significant desaturations; thus, they cannot be detected by the standard NOx [4,16,18].

The aim of the present study is to apply advanced NOx signal analysis for developing novel indices that may improve the diagnostic performance of this method. We hypothesize that children who undergo adenotonsillectomy for clinically suspected OSAS will present a significant improvement in oximetry parameters three months after surgery and, thus, can serve as self-controls for assessing the diagnostic performance of both standard and novel NOx indices.

## 2. Materials and Methods

### 2.1. Study Design and Population

This is a prospective observational study that was performed between June 2019 and December 2021 in the Department of Otorhinolaryngology of the ‘Karamandaneio’ Children’s Hospital of Patras, Greece, which is the referral pediatric otorhinolaryngology center for the Region of Western Greece (approximately 1,000,000 inhabitants). The study was originally planned to be completed in December 2020; however, a 12-month extension was required due to the COVID-19 pandemic.

Children eligible to participate were those over 2 years of age who were scheduled for adenotonsillectomy due to (**a**) clinically significant OSAS identified by a standardized pediatric sleep questionnaire (PSQ) [19] score >0.35, (**b**) significant tonsillar hypertrophy upon examination of the oropharynx, defined as tonsillar size > 2 in Friedman’s scale [20], and (**c**) significant adenoid hypertrophy, as estimated during endoscopic examination of the nasopharynx and/or by an adenoid-to-nasopharynx ratio [21] >0.75 on the lateral soft tissue neck radiograph [22], i.e., the standard criteria that determine the decision to perform adenotonsillectomy in our facility due to lack of PSG. Children with craniofacial deformities, neuromuscular disorders, or chromosomal abnormalities were excluded from this study.

This study was conducted in accordance with the Declaration of Helsinki and approved by the Institutional Scientific Board of ‘Karamandaneio’ Children’s Hospital (act no. 5841/23.5.2019). Written informed consent was obtained from the parents of all participants before enrollment.

### 2.2. Study Protocol

Eligible children were assessed before adenotonsillectomy (study visit 1) and three months later (study visit 2).

Study visit 1 was performed at the time of preoperative assessment on the day before the scheduled operation. All children were examined by a consultant otorhinolaryngologist and, apart from the standard clinical and laboratory testing, the PSQ was re-evaluated, and the size of the tonsils and adenoids was confirmed. The parents were informed in detail about the aims of the present study, and those who consented to participate were asked to attend a short (10 min) basic course on NOx (principle of operation, device handling, sensor positioning and fixing, types and significance of alarms) by one of the investigators. NOx studies were performed the night before adenotonsillectomy in a dedicated single-bed room of the otorhinolaryngology ward.

In study visit 2, the participants performed NOx at their homes three months (±1 week) after adenotonsillectomy. The pulse oximetry device was installed by one of the investigators who also repeated the presentation of the short basic course of NOx to the parents. The parents were given the possibility to contact the researcher if any problem arose during the sleep study; the device was collected by the researcher on the following day.

### 2.3. NOx Recordings and Analysis

NOx measurements were performed using a Nonin 7500 pulse oximeter (Nonin Medical Inc, Plymouth, MN, USA) utilizing a 4-beat (or ≤3 s) exponential SpO_2_ averaging algorithm, with a pediatric single-use cloth sensor (6000C series, Nonin Medical Inc) placed on the index, middle, or ring finger, as appropriate. The parents or guardians were asked to record the exact time the child fell asleep and woke up; they were instructed to also record events, such as snoring, increased work of breathing, arousal, etc., on a special observation sheet. The minimum duration of the study was set to 6 h.

#### 2.3.1. Standard NOx Analysis

The recordings were initially reviewed and analyzed using nVision software (Nonin Medical Inc). The parts at the beginning and end of the recording corresponding to the awake time according to the observation sheet were excluded. No further manual editing was performed; poor signal quality SpO_2_ data (e.g., due to motion) were automatically excluded by the software. The following parameters were recorded: study duration, percentage (%) of artifacts, average SpO_2_ value, ODI4 (times per hour the SpO_2_ dropped by ≥4% from baseline and for at least 10 s), and ODI3 (times per hour the SpO_2_ dropped by ≥3% from baseline and for at least 10 s). Recordings with an artifact rate >2.5% were excluded.

NOx tracings were inspected by an investigator who was unaware of the clinical status of the participant, and McGill oximetry score (MOS) was assigned as follows [16,23]:

- MOS 1 (non-diagnostic or inconclusive): <3 desaturation clusters, without desaturation <90%;
- MOS 2 (mild OSAS): ≥3 desaturation clusters, with ≥3 desaturations <90% and no desaturation <85%;
- MOS 3 (moderate OSAS): ≥3 desaturation clusters, with ≥3 desaturations <85% and no desaturation <80%;
- MOS 4 (severe OSAS): ≥3 desaturation clusters, with ≥3 desaturations <80%.

A desaturation event was defined as a SpO_2_ drop of ≥4% from baseline and for at least 10 s; a cluster of desaturations was defined as ≥5 desaturations within a 10–30 min study period.

#### 2.3.2. Novel NOx Indices

**Figure 1:**
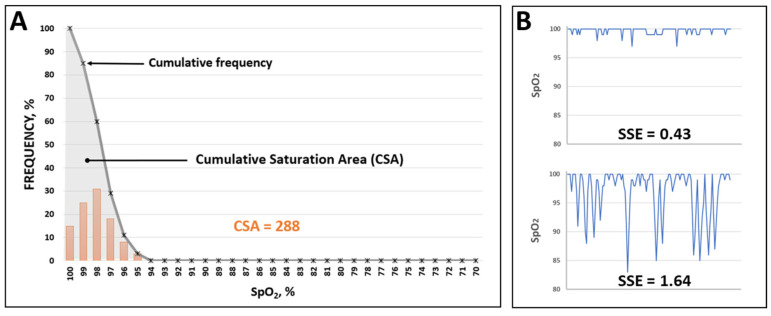
(**A**) Calculation of CSA. (**B**) An example of a NOx trace with decreased SSE (upper) and one with increased SSE (lower). Note how the more regular the signal, the lower the entropy and vice versa.

Raw NOx data (4 s resolution) were extracted and analyzed in MatLab (Mathworks Inc, Boston, MA, USA), and two novel NOx indices were calculated:

- Cumulative saturation area (CSA) (Figure 1A). The frequency distribution plot of SpO_2_ values was constructed, and the cumulative frequency at each SpO_2_ data point was obtained. The CSA was calculated as the area under the cumulative frequency line (i.e., the line that connects cumulative frequencies) (Figure 1A). Theoretically, this dimensionless parameter reflects the overall spread of SpO_2_ values and increases as the rate of lower SpO_2_ values increases. The minimum expected CSA value (i.e., in the case that all SpO_2_ values are equal to 100%) is 100.
- SpO_2_ sample entropy (SSE). Entropy is a well-established mathematical metric to describe the regularity or stability of non-stationary time series such as biological signals [24]. In this regard, an SpO_2_ time series without frequent and/or significant fluctuations from the baseline (i.e., desaturations) is expected to be more regular; this is reflected by a decreased entropy and vice versa [25] (Figure 1B). Among numerous entropy algorithms, SampEn has the advantage of being independent of data length and easier to implement [24]. SampEn was calculated according to the source code provided by Delgado-Bonal and Marshak [24].

CSA: cumulative saturation area, NOx: nocturnal oximetry, and SSE: SpO_2_ sample entropy.

Characteristic examples of CSA and SSE calculation are presented in Figure S1. In addition to the above parameters, the standard deviation (SD) of the SpO_2_ values was computed and recorded.

### 2.4. Statistics

Data are presented as mean ± SD (median; range) or number of cases (%). Changes in NOx indices after adenotonsillectomy were assessed with the Wilcoxon matched-pairs signed-rank test. Receiver operating characteristics (ROCs) analysis was applied to evaluate the diagnostic characteristics of NOx parameters; post-adenotonsillectomy NOx tracings were considered as normal controls. The appropriate diagnostic cutoffs were calculated according to the highest Youden’s index (i.e., sensitivity plus specificity). Spearman’s correlation was applied to assess the relationships among key participants’ characteristics and NOx parameters before adenotonsillectomy. All analyses were performed with IBM SPSS v.28 (IBM Corp, Armonk, NY, USA).

## 3. Results

**Figure 2:**
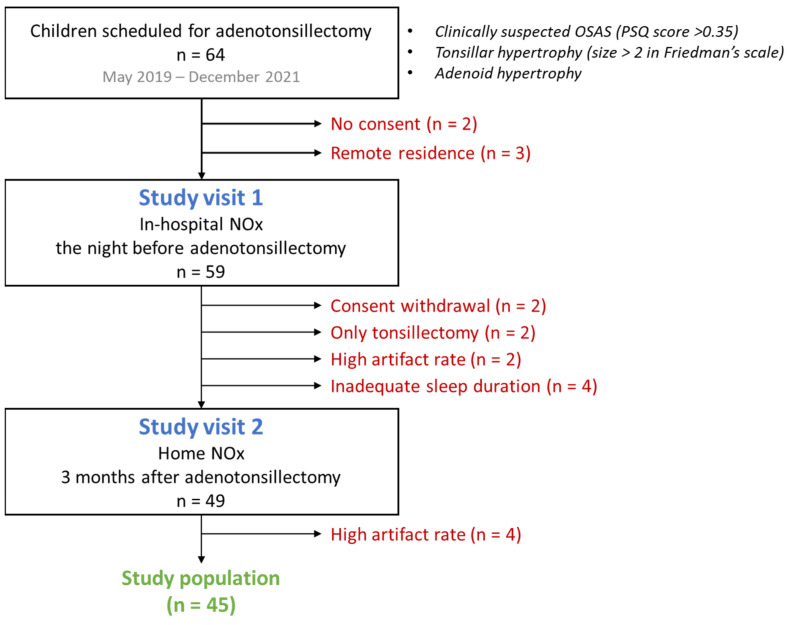
Study flow diagram.

Between May 2019 and December 2021, 64 eligible children were identified. The parents of four children did not consent to participate (two withdrew their consent after the operation), while in three cases, the family resided in a remote region (more than 100 km away from the city of Patras), and the visit at home (study visit 2) was deemed impractical. Of the remaining children, two underwent only tonsillectomy despite the initial plan for combined adenotonsillectomy, six had NOx recordings with a rate of artifact ≥2.5% (two on study visit 1 and four on study visit 2; artifact range 3.2 to 12.8%), and in four cases, the sleep duration was less than 6 h (range 4.9 to 5.7 h). Therefore, the final study population consisted of 45 children. A study flow diagram is shown in Figure 2.

The age of the participants ranged from 3 to 10 years, and 55.6% of them were boys. The characteristics of the study population and the NOx parameters before and after adenotonsillectomy are presented in Table 1. The duration of recording was 6.7 ± 0.5 h (median, 6.7; range, 6–7.4 h) before and 6.4 ± 0.4 h (median, 6.5; range, 6–7.1 h) after adenotonsillectomy; the artifact rate was 0.5 ± 0.4% (median, 0.4; range, 0.1–1.8%) and 0.4 ± 0.2% (median, 0.4; range, 0.1–1.9%), respectively. The MOS was inconclusive or not diagnostic (MOS 1) in 37.8% of the children.

**Figure 3:**
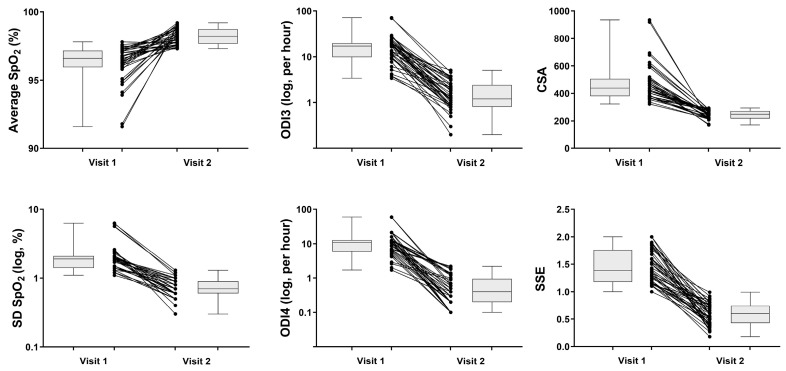
NOx parameters before (visit 1) and three months after adenotonsillectomy (visit 2) in our cohort (n = 45). The box plots depict the median, 25–75th percentile range, and range. Mean ± SD (median; range) is also presented. Comparisons were performed with Wilcoxon matched-pairs signed-rank test. All differences are significant at *p* < 0.001.

There were significant changes (*p* < 0.001) in all NOx parameters after adenotonsillectomy; the average SpO_2_ increased, while SD SpO_2_, ODI3, ODI4, CSA, and SSE decreased (Figure 3).

The diagnostic characteristics of NOx parameters are presented in Table 2. A MOS > 1 presented excellent specificity but only moderate sensitivity in diagnosing clinically significant upper airway obstruction. Conversely, ODI3 (at a cutoff of >3.6 events per hour) and ODI4 (>2.2 events per hour) were both highly sensitive and specific, while the diagnostic characteristics of CSA (at a cutoff of >293) and SSE (at a cutoff of >0.99) were excellent (Table 2).

**Figure 4:**
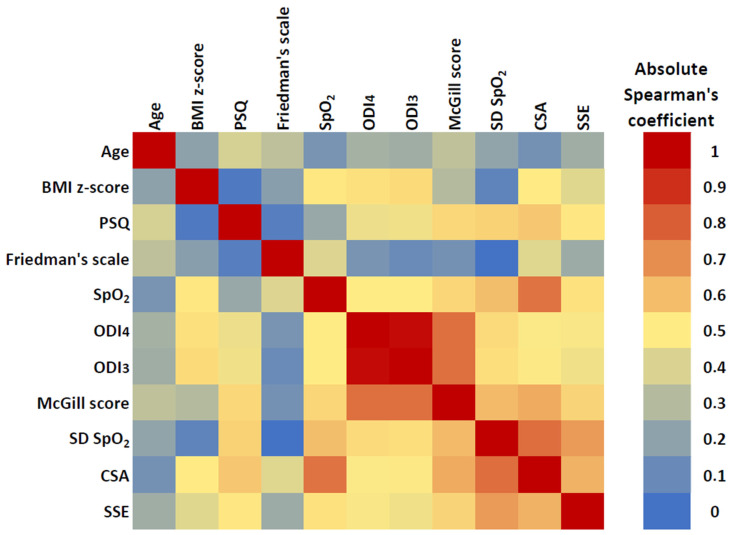
Heatmap showing the magnitude of the correlation between participants’ characteristics and NOx parameters before adenotonsillectomy. The color variation from light blue (‘cold’) to deep red (‘hot’) relates to the absolute value of Spearman’s correlation coefficient (exact coefficient values are presented in Table S1).

The correlation between participants’ characteristics and NOx parameters before adenotonsillectomy is presented in Figure 4, while the exact correlation coefficient values are given in Table S1. ODI3 and ODI4 were highly correlated (r = 0.975); both presented a good correlation with MOS (r = 0.710 and 0.707, respectively) but only a modest correlation with the PSQ score (r = 0.353 and 344, respectively). CSA had a good correlation with the average SpO_2_ (r = −698) and the SD of SpO_2_ (r = 0.713) and a modest correlation with MOS (r = 0.548) and PSQ score (r = 0.478). The correlation of SSE with all the above parameters was modest, similar to the correlation between SSE and CSA (r = 0.533) (Figure 4, Table S1).

## 4. Discussion

In this paper, by applying advanced SpO_2_ signal analysis, we developed and presented two novel NOx indices, namely, the CSA and SSE. The former (CSA) reflects the distribution of SpO_2_ values throughout the NOx recording, and the latter (SSE) reflects the regularity of the SpO_2_ time series. Subsequently, we assessed the diagnostic performance of CSA and SSE, together with the performance of MOS, ODI4, and ODI3 in a cohort of children who underwent adenotonsillectomy for clinically suspected OSAS, considering the NOx recordings at three months post-surgery as self-controls of those performed before the operation. As expected, all the standard NOx indices improved significantly post-adenotonsillectomy, and they normalized. We found that the MOS presented moderate discriminatory ability with a high false negative rate, while the ODI3 and ODI4 performed significantly better but still with false negative and false positive results. On the other hand, the CSA and SSE had excellent discriminatory characteristics with 100% sensitivity and specificity, thus, indicating that these novel indices hold promise in improving the diagnostic ability of NOx in children suspected of OSAS.

Currently, NOx is considered a reasonable alternative for OSAS diagnosis when PSG is not available [15,16]. The MOS, which relies on the identification of desaturation clusters (i.e., repeated events of SpO_2_ drop by ≥4% from the baseline within a period of 10–30 min), represents the most popular OSAS-related outcome [1]. A positive score (i.e., MOS ≥ 2) performs excellent in identifying children with moderate-to-severe OSAS, but its false negative rate is high [16]; in a previous study [14], 36.2% of children with inconclusive MOS (MOS = 1) had an AHI of >5 events per hour, while 41.8% of those with AHI >5 had an inconclusive MOS in a more recent report [26]. In our study, the inconclusive MOS rate before adenotonsillectomy was 37.8% (Table 1), thus, confirming that MOS per se may lead to the significant misclassification of children with moderate-to-severe OSAS as having mild or even no disease [16].

The ODI refers to the times per hour the SpO_2_ drops by ≥4% (ODI4) or ≥3% (ODI3) from the baseline. A systematic review concluded that an ODI4 of >2 events per hour is indicative of mild OSAS, while higher values are characteristic of more severe disease [16]. The herein-reported cutoff of >2.2 events per hour (Table 2) is, thus, in line with the existing literature [16]. Nevertheless, given the overlap between the lower centiles of ODI4 in children with OSAS and the upper centiles of their asymptomatic counterparts [27,28], the definition of an accurate cutoff with balanced diagnostic performance (i.e., sensitivity vs. specificity) has proven to be difficult [1,16]. Therefore, many expert panels have proposed that the ODI3 is more appropriate to describe NOx abnormality [7,29]; an ODI3 of ≥3.5 events per hour is considered predictive of OSAS [28], while this cutoff performs similarly to AHI > 5 in detecting children who are more likely to benefit from adenotonsillectomy [18]. In our study, an ODI3 of >3.6 events per hour had the best diagnostic performance (sensitivity and specificity >90%, Table 2), thus, corroborating the above reports [18,28]. However, the ODI3 was falsely negative in one participant before adenotonsillectomy and falsely positive (i.e., >3.6) in four children (8.9%) after the operation. Although lower than those reported in studies focusing on moderate-to-severe OSAS [18,26], these figures highlight the risk of misclassification when ODI3 is applied as the sole predictor.

Despite their widespread use, both MOS and ODIs have the disadvantage of being user-defined; the magnitude (≥3% or ≥4%) and duration (≥10 s or other) of SpO_2_ drop, used to define desaturations, and the rather variable time frame (10–30 min) and the number of events (≥5), used to trace desaturation clusters, have emerged from expert opinions [7,29] rather than experimental or clinical evidence. In contrast, the calculation of the herein-introduced indices, CSA and SSE, relies solely on the raw oximetry data without the need to apply predefined SpO_2_ or time-related cutoffs.

This study is the first to apply and evaluate the CSA and SSE as NOx outcome measures. The CSA reflects the distribution of SpO_2_ values (Figure 1A, Figure S1) and is dependent on their average and spread; the lower the mean SpO_2_ and/or the higher the SD of SpO_2_ values, the higher the CSA. Thus, CSA may be a sensitive measure of intermittent hypoxemias that characterize the hypopneas and apneas in OSAS [4,16]. The SSE, on the other hand, is a composite mathematical index that reflects the overall regularity of the SpO_2_ time series [24]; indeed, frequent and/or significant SpO_2_ fluctuations, such is the case with the OSAS-related desaturations, result in high SSE and vice versa [25] (Figure 1B). In our study, the CSA presented a good correlation with both the average and the SD of SpO_2_, while the SSE was significantly correlated only with the SD of SpO_2_ (Figure 4). The correlation between CSA and SSE was modest, suggesting that the two indices reflect different aspects of the same physiological process, which results in intermittent hypoxemias.

Both CSA and SSE can be easily computed by straightforward algorithms, under the condition that the raw SpO_2_ data are stored and available. However, their calculation may be influenced by the technical characteristics of the pulse oximeter, such as the averaging time, the algorithms used to detect motion artifacts and poor signal quality, and the resolution (i.e., the frequency) of SpO_2_ data storage [30]. The herein-used Nonin 7500 oximeter (Nonin Medical Inc) utilizes a four-beat (corresponding to ≤3 s) exponential averaging algorithm and incorporates an advanced pulse-by-pulse filtering technology (PureSAT^®^) that enables the proper recognition and elimination of erroneous SpO_2_ values due to motion or low perfusion. Although the above characteristics are generally in line with the recommended technical standards [29], shorter averaging times, different signal extraction algorithms, and higher data resolution may influence CSA and SSE values.

The lack of PSG data is the most important limitation of the present study. PSG is the gold standard for OSAS diagnosis in children [1], even though it is laborious, and the availability of pediatric sleep laboratories is limited [12,13,16]. Our study recruited children with high PSQ scores and significant adenotonsillar hypertrophy, all of whom were free of sleep-disordered breathing 3 months after adenotonsillectomy, and thus, they served as self-controls. However, given the lack of PSG testing, the complete resolution of hypopneas and/or apneas after adenotonsillectomy cannot be confirmed. Indeed, in the Childhood Adenotonsillectomy Trial [9], the PSG parameters did not normalize in 21% of the children 7 months after surgery, while almost 55% of the participants presented an incomplete resolution of intermittent hypoxemias at 3 months post-adenotonsillectomy in a more recent study [18]. In our cohort, all NOx parameters improved remarkably after the operation (Figure 3), while both ODI3 and ODI4 at visit 2 were within the normal range (i.e., lower than the reported 90^th^ centile) [27]; therefore, it is highly unlikely that our participants presented significant residual disease at 3 months after adenotonsillectomy. In addition, the lack of PSG did not permit us to account for different degrees of OSAS severity, which represents an important aspect of the relevant research [1]. Finally, these novel NOx indices are subject to the limitations of oximetry as a screening tool for OSAS; indeed, milder episodes of upper airway obstruction may cause disordered breathing symptoms (snoring, arousals, etc.) but not intermittent hypoxemias and, therefore, remain undetectable [4,16].

The originality of this work resides in applying—for the first time—advanced SpO_2_ signal analysis to introduce two novel NOx indices, namely, the CSA and SSE, which can be easily computed and theoretically lack the disadvantages of the standard NOx parameters. It should be noted, however, that this was a proof-of-concept study that only intended to present a preliminary validation of these novel NOx indices. In addition to the clinical evaluation of the CSA and SSE in larger, PSG-based studies, future research should also focus on the determination of minimum technical requirements for the calculation of these novel indices, as well as on the way such technicalities may influence their values. Such studies should also explore the utility of CSA and SSE in the context of machine learning approaches, which, by combining symptoms, clinical examination, and NOx indices, may assist in the diagnosis of OSAS without the need for PSG [31]. Finally, composite indices that explore the non-linear dynamics of the oximetry signal, such as the SSE, hold promise for the improvement in automated NOx analysis and the increase in the diagnostic performance of the method [32].

## 5. Conclusions

When PSG is not available, standard NOx parameters, such as the MOS and the oxygen desaturation indices, may be used to detect children with suspected OSAS who will benefit from adenotonsillectomy. However, because these parameters depend on user-defined cutoffs, their diagnostic performance is limited by the significant false negative and/or false positive rates. The herein-introduced novel NOx indices, namely, CSA and SSE, overcome the above limitations and present superior diagnostic characteristics. Future research should focus on a more detailed clinical evaluation of CSA and SSE as stand-alone and/or add-on predictors in the context of relevant clinical profiles. By improving the automated NOx analysis and increasing the diagnostic performance of the method, these novel parameters may also be incorporated into machine learning approaches which, by combining clinical characteristics and NOx indices, may assist in the accurate diagnosis of OSAS without the need for PSG.

## Figures and Tables

**Table 1:** Characteristics of the study population (n = 45) and NOx parameters pre- and three months post-adenotonsillectomy.

Male Sex, n (%) Age, years	25 (55.6)
	4.8 ± 1.8 (5; 3–10)
BMI z-score	0.87 ± 1.02 (0.98; –1.2–2.8)
PSQ score pre	0.53 ± 0.09 (0.55; 0.5–0.72)
PSQ score post	0.20 ± 0.06 (0.23; 0.0–0.37)
SpO_2_ pre, %	96.2 ± 1.4 (96.6; 91.6–97.8)
SpO_2_ post, %	98.2 ± 0.6 (98.2; 97.3–99.2)
ODI4 pre, per hour	12.1 ± 11.6 (10.9; 1.7–59.4)
ODI4 post, per hour	0.6 ± 0.6 (0.4; 0.1–2.2)
ODI3 pre, per hour	17.8 ± 13.4 (17; 3.4–71.3)
ODI3 post, per hour	1.7 ± 1.2 (1.2; 0.2–5.1)
MOS pre	1.93 ± 0.94 (2; 1–4)
MOS 1 (not diagnostic), n (%)	17 (37.8)
MOS 2 (mild OSAS), n (%)	18 (40)
MOS 3 (moderate OSAS), n (%)	6 (13.3)
MOS 4 (severe OSAS), n (%)	4 (8.9)
MOS post	1 ± 0.0 (1; 1–1)
SD SpO_2_ pre, %	2.1 ± 1.3 (1.9; 1.1–6.3)
SD SpO_2_ post, %	0.7 ± 0.2 (0.6; 0.3–1.1)
CSA pre	473 ± 136 (440; 322–935)
CSA post	245 ± 30 (248; 171–293)
SSE pre	1.46 ± 0.30 (1.39; 1.00–2.00)
SSE post	0.58 ± 0.19 (0.60; 0.18–0.99)

Data presented as mean ± SD (median; range) unless stated otherwise. NOx: nocturnal oximetry, BMI: body mass index, PSQ: pediatric sleep questionnaire, SpO_2_: average oxyhemoglobin saturation by pulse oximetry, ODI4: oxygen desaturation ≥4% index, ODI3: oxygen desaturation ≥3% index, MOS: McGill oximetry score, OSAS: obstructive sleep apnea syndrome, SD: standard deviation, CSA: cumulative saturation area, and SSE: SpO_2_ sample entropy.

**Table 2:** Diagnostic characteristics of selected NOx parameters.

Parameter	AUC (95% CI)	Cutoff ^1^	Se %	Sp %
MOS	0.811 (0.715–0.886) ^2^	>1	62.2	100
ODI3	0.994 (0.948–0.997)	>3.6 per hour	97.8	91.1
ODI4	0.990 (0.945–0.992)	>2.2 per hour	93.3	95.6
CSA	1.000 (0.959–1.000)	>293	100	100
SSE	1.000 (0.959–1.000)	>0.99	100	100

Receiver operating characteristics (ROCs) analysis. ^1^ according to the highest Youden’s index (i.e., sensitivity plus specificity). ^2^ significantly lower (*p* < 0.001), as compared to the other parameters. AUC: area under the ROC curve, Se: sensitivity, and Sp: specificity.

## Data Availability

The data presented in this study are available on reasonable request from the corresponding author.

## Supplementary Materials

The following supporting information can be downloaded at: https://www.mdpi.com/article/10.3390/children10030453/s1, Figure S1: Examples of CSA calculation with the respective NOx traces from which they have derived; Table S1: Correlation between participants’ characteristics and NOx parameters before adenotonsillectomy.
