# Supplementary material for: Novel Indices to Improve the Diagnostic Ability of Nocturnal Oximetry in Children with OSAS
Source: Children (Basel). 2023 Feb 25;10(3):453. doi: 10.3390/children10030453 (PMC10047685; doi:10.3390/children10030453)
Supplement: Supplementary file 1 [file children-10-00453-s001.zip › Figure S1_R1.pdf]

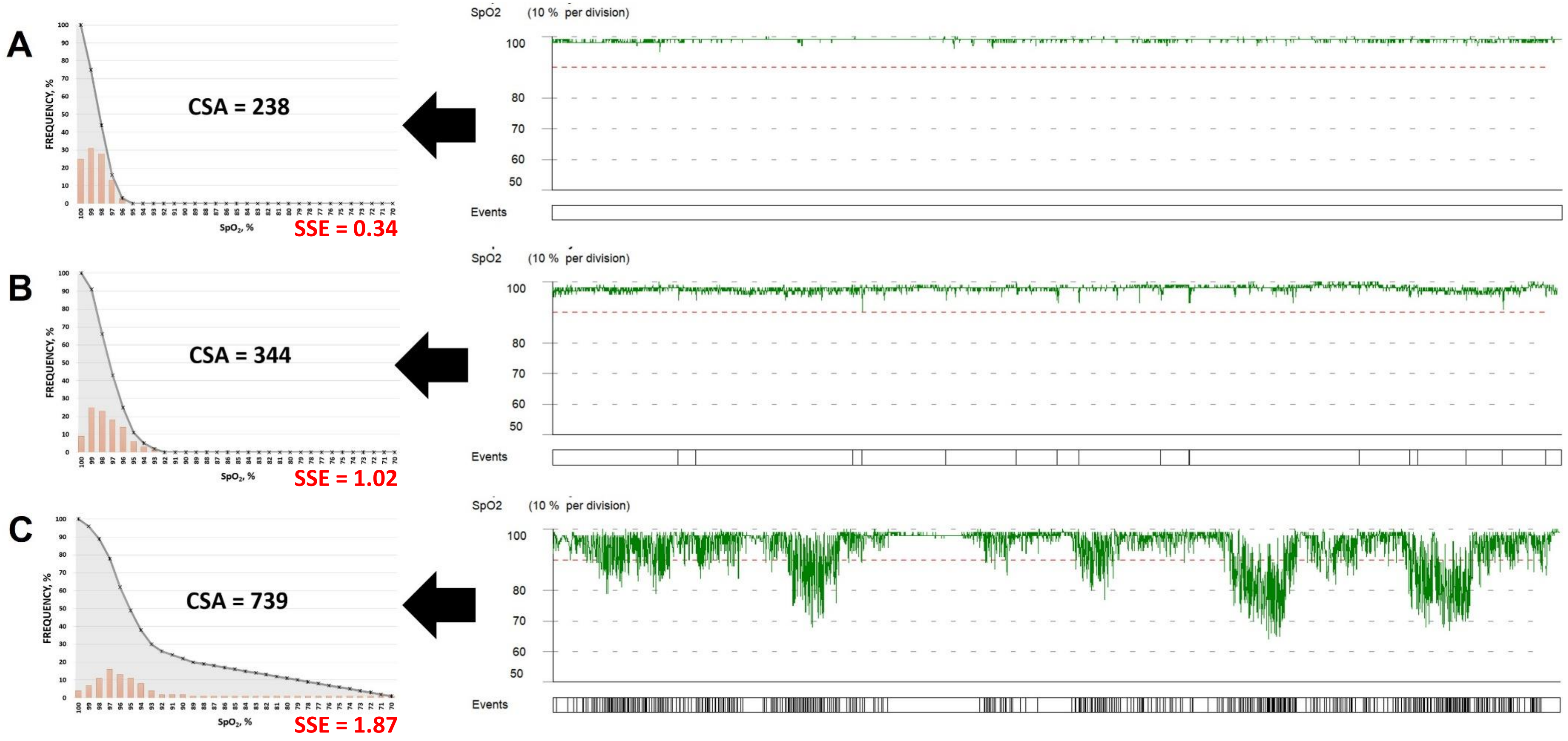

Figure S1. Examples of CSA calculation with the respective NOx traces from which they have derived. (A) Post-adenotonsillectomy recording (MOS = 1, ODI_3 = 0 per hour). The CSA is low. (B): Pre-adenotonsillectomy recording (MOS = 1, ODI_3 = 2.9 per hour). The CSA is relatively high (>300) despite the normal MOS and ODI_3. (C) Pre-adenotonsillectomy recording (MOS = 4, ODI_3 = 56.9 per hour). Note how the CSA extends 'on the right' due to the presence of low SpO_2 values; the CSA is abnormal. The respective SSE values are also shown (in red).

CSA: cumulative saturation area, NOx: nocturnal oximetry, MOS: McGill oximetry score, ODI_3: oxygen desaturation ≥ 3% index, SSE: SpO_2 sample entropy
