# Supplementary material for: Novel Indices to Improve the Diagnostic Ability of Nocturnal Oximetry in Children with OSAS
Source: Children (Basel). 2023 Feb 25;10(3):453. doi: 10.3390/children10030453 (PMC10047685; doi:10.3390/children10030453)
Supplement: Supplementary file 1 [file children-10-00453-s001.zip › Table S1.pdf]

Table S1. Correlation among participants' characteristics and NOx parameters before adenotonsillectomy

	Age	BMI z-score	PSQ	Friedman scale	SpO_2	ODI4	ODI3	MOS	SD SpO_2	CSA	SSE
**Age**	1.000	0.151	–0.297	0.249	–0.108	–0.199	–0.189	–0.251	–0.158	0.100	0.188
**BMI z-score**	0.151	1.000	0.024	–0.139	–0.397	0.411	0.429	0.229	0.056	0.386	0.320
**PSQ**	–0.297	0.024	1.000	0.040	–0.172	0.344	0.353	0.437	0.448	0.478	0.395
**Friedman scale**	0.249	–0.139	0.040	1.000	0.311	0.108	0.079	–0.099	0.002	–0.318	0.182
**SpO_2**	–0.108	–0.397	–0.172	0.311	1.000	–0.382	–0.382	–0.439	–0.502	–0.698	–0.410
**ODI4**	–0.199	0.411	0.344	0.108	–0.382	1.000	0.975	0.707	0.427	0.375	0.368
**ODI3**	–0.189	0.429	0.353	0.079	–0.382	0.975	1.000	0.710	0.417	0.377	0.353
**MOS**	–0.251	0.229	0.437	–0.099	–0.439	0.707	0.710	1.000	0.511	0.548	0.446
**SD SpO_2**	–0.158	0.056	0.448	0.002	–0.502	0.427	0.417	0.511	1.000	0.713	0.595
**CSA**	0.100	0.386	0.478	0.318	–0.698	0.375	0.377	0.548	0.713	1.000	0.533
**SSE**	0.188	0.320	0.395	0.182	–0.410	0.368	0.353	0.446	0.595	0.533	1.000

Data represent Spearman's correlation coefficients

NOx: nocturnal oximetry, BMI: body mass index, PSQ: pediatric sleep questionnaire, SpO2: average oxyhemoglobin saturation by pulse oximetry, ODI4: oxygen desaturation ≥ 4% index, ODI3: oxygen desaturation ≥ 3% index, MOS: McGill oximetry score, SD: standard deviation, CSA: cumulative saturation area, SSE: SpO2 sample entropy
